# Fractional-Order Epidemic Model for Measles Infection

**DOI:** 10.1155/2024/8997302

**Published:** 2024-10-10

**Authors:** Philip N. A. Akuka, Baba Seidu, Eric Okyere, Stephen Abagna

**Affiliations:** ^1^Department of Mathematics, Bongo Senior High School, Bongo, Ghana; ^2^Department of Mathematics, School of Mathematical Sciences, C. K. Tedam University of Technology and Applied Sciences, Navrongo, Ghana; ^3^Department of Mathematics and Statistics, University of Energy and Natural Resources, Sunyani, Ghana; ^4^Department of Mathematics, Sirigu Integrated Senior High School, Sirigu, Ghana

## Abstract

In this study, a nonlinear dynamic SEVIQR measles epidemic model is constructed and analyzed using the novel Caputo fractional-order derivative operator. The model's existence and uniqueness are established. In addition, the model equilibria are determined, and the novel Jacobian determinant method recently constructed in the literature of epidemiological modeling of infectious diseases is applied to determine the threshold quantity, *ℛ*_0_. Furthermore, we construct appropriate Lyapunov functions to establish the global asymptotic stability of the disease-free and endemic equilibrium points. Finally, the numerical solution of the model is executed employing the efficient and widely known Adams-type predictor-corrector iterative scheme, and simulation is conducted to investigate the impact of memory index and diverse preventive measures on the occurrence of the disease. Numerical simulation of the model indicates that quarantine, vaccination, and treatment can reduce the numbers of infectious and exposed populations, thereby controlling the disease. Therefore, it is recommended that the government provide financial assistance for vaccine distribution.

## 1. Introduction

Measles, an infectious disease resulting from the measles virus, belongs to the paramyxoviridae class and manifests symptoms such as fever, characteristic rash, cough, coryza, and conjunctivitis. It is highly contagious and spreads through respiratory droplets and contact with contaminated surfaces. The latent period from exposure to prodromal symptoms averages 11 to 12 days. However, it starts with a high fever for about 10–12 days when an individual encounters an infected person or surfaces [1]. Measles continues to pose a significant public health challenge worldwide, particularly in nations with lower- and middle-income levels, though safe and affordable vaccines are accessible [2]. According to the World Health Organization (WHO), in 2020, the number of children missing essential childhood vaccinations through routine health services reached its highest level since 2009, affecting 23.5 million children, an increase of 3.8 million compared to 2019. Furthermore, the global incidence of measles cases has surged by approximately 79% in the initial two months of 2022 compared to the corresponding period in 2021 [3]. The northern region of Ghana recorded approximately 120 new cases of measles as of February 2023 due to the shortage of essential vaccines, according to the Paediatric Society of Ghana. The Ghana Health Service (GHS) attributes the vaccine shortage to the depreciation of the Ghana Cedi, which affects the routine vaccination program for babies. This program includes tetanus, Bacillus Calmette–Guérin (BCG), whooping cough, tuberculosis (TB), and oral polio vaccine (OPV) [4].

Mathematical modeling is a valuable approach to understand the patterns of how infectious diseases spread. Utilizing mathematical tools allows for a deeper understanding of various infectious diseases. Throughout the existing body of the literature, interdisciplinary efforts involving epidemiologists, mathematicians, ecologists, and statisticians have been undertaken to elucidate the spread of diseases such as measles, influenza, HIV/AIDS, cholera, COVID-19, malaria, Ebola, dengue fever, Zika virus, and tuberculosis. These efforts often involve the construction of mathematical models, typically expressed as sets of nonlinear autonomous systems of first-order ordinary differential equations (ODEs), to effectively understand and suggest appropriate control strategies. Mathematical modeling has gained popularity in the field of infectious disease research. Using this technique, researchers have the ability to create a set of equations to examine the dynamics of various infectious diseases, providing valuable insights into their behavior. Over the years, numerous researchers have investigated various infectious diseases and developed mathematical models to describe the spread of epidemics. These models have undergone stability analysis (see, for example, [5–14]), which aids in managing and preventing the spread of infectious diseases in everyday life. The modeling of epidemic models has proven to be useful in both academic and daily life contexts. Wallinga et al. [15] conducted a study focused on measles epidemics within a population with high vaccination coverage. Their findings pointed to a critical factor in measles outbreaks: the presence of susceptible children. Based on their research, the authors advocated for the implementation of an extensive vaccination campaign at a high level to effectively preempt measles epidemics. In another study, Rehman et al. [16] presented a study that described the physical solution of the SIQR model for measles spread under the effect of natural delay. James et al. [17] proposed a model which investigated the impact of vaccination and hospitalization on the dynamics of measles. Their findings showed that the combined control strategies reduce the peak of infection faster than the single control strategy. Furthermore, Fakhruddin et al. [18] formulated an SIHR epidemic model of measles involving vaccination. Their results showed that providing treatment accesses easier for infected persons is better than vaccinating when an outbreak occurs. Kuddus et al. [19] also studied a compartmental mathematical model to examine the effect of double- dose vaccination on measles dynamics in Bangladesh.

Agur et al. [20], in a study, introduced a deterministic age-structured model in their experimental investigation. They examined the efficacy of a pulse strategy, characterized by periodic vaccination efforts. The research demonstrated that the pulse strategy was indeed effective. The study also explored less complex modes without age structure to clarify the essential traits of the suggested strategy. The findings consistently supported the conclusion that the pulse strategy effectively achieved its intended goals [20]. Cutts et al. [21], in their study, reached the conclusion that mathematical models that focus on school-age groups (age-structured models) provide a superior approach compared to global mass-action models when it comes to understanding and studying measles [22]. Roberts and Tobias developed a measles model with the primary goal of determining the best timing for measles-mumps-rubella (MMR) immunization. This research was conducted in New Zealand, focusing on the critical issue of when to administer MMR immunization to predict and prevent measles outbreaks. Their approach involved using a mathematical model to replicate how measles spreads within the population and to evaluate the efficacy of different vaccination approaches. The study's key finding was that the ideal timing for MMR immunization hinges on both the age distribution of the susceptible population and the timing of the most recent epidemic. Consequently, the study concluded that the most effective timing for MMR immunization should be tailored to the age distribution of susceptible individuals and the occurrence of previous outbreaks [23]. Many models in the scientific literature have either been introduced or adapted to enhance our comprehension of how measles epidemics spread and evolve [24–30]. Nonetheless, it is important to note that all these models rely on classical derivatives and integrals, which can impose limitations on the flexibility in choosing the order of the differential equations. Many researchers have turned to a relatively new and widely embraced branch of analyzing mathematical models known as the fractional calculus to recognize the limitations associated with models employing local classical derivatives [31]. This approach involves the use of nonlocal differential operators with memory effects, which prove valuable in modeling physical and natural phenomena displaying unusual behaviors and nonlocal dynamics. The inclusion of fractional-order (FO) derivatives in models to study measles is motivated by the recognition that the dynamics of epidemiological processes in human populations is notably shaped by memory [32]. The various types of fractional operators have been introduced to gain deeper understanding of how these models behave (some examples of these operators include Riemann–Liouville, Hadamard, Katugampola, Caputo, Caputo–Fabrizio, Atangana–Baleanu, Atangana–Gomez, Atangana-Koca, Atangana beta-derivative, truncated *𝕄*-derivative, Atangana biorder, and numerous others [33]). Each of these operators possesses distinct advantages and disadvantages relative to others [33, 34].

For instance, Riemann–Liouville fractional operators demand fractional-order conditions for solving the mathematical models under investigation, adding complexity to the analysis. On the contrary, the Caputo fractional operator eliminates this restriction, enabling the use of initial conditions that involve derivatives of integer order, which have distinct and easily understandable physical interpretations [32]. In this current investigation, we utilize the Caputo fractional operator, which exhibits nonlocal properties and a singular kernel, to effectively model the dynamics of measles transmission. Various categories of infectious diseases and associated practical challenges have been more recently analyzed and represented using the Caputo fractional operator (see, for example, [9, 35–42]). These nonlocal fractional operators have demonstrated to be highly effective not only in modeling infectious diseases but also in enhancing the performance of a wide range of physical and engineering systems [43–46]. Deterministic compartmental models have been developed to investigate alcohol addiction utilizing Caputo-based fractional and fractal-fractional derivative operators [47]. The authors in [48] employed the Caputo fractional derivative operator to construct and analyze a nonlinear dynamic epidemiological model that investigated the transmission patterns of the recent monkeypox outbreak in the United States. In another study, the authors in [49] developed autonomous and nonautonomous dynamic epidemic models characterized by Caputo fractional-order derivatives to investigate the transmission dynamics and control of Nipah virus infection. In addition, numerous researchers have utilized dynamic models based on fractional calculus to mathematically describe the transmission dynamics of various diseases such as polycystic ovarian syndrome [8], Ebola virus infection [50–53], syphilis [54–56], and cancer [57–60].

Abboubakar et al. [61] proposed a new epidemiological system for the measles epidemic using Caputo fractional derivative with a memory effect. Ogunmiloro et al. [62] studied the transmission dynamics of measles with double vaccination dose, treatment, and two groups of measles-infected and measles-induced encephalitis-infected humans with relapse under the fractional Atangana–Baleanu–Caputo (ABC) operator. Also, Abboubakar et al. [61] modified an existing compartmental model of measles transmission that accounted for vaccinations and hospitalizations by substituting the integer derivative with the Caputo derivative. This allowed them to incorporate fractional calculus into the model and better capture the dynamics of the system. Their aim was to investigate the impact of memory on the model, which was developed based on the transmission phenomena of measles. Almeida and Qureshi's [63] study focuses on fractionalizing a measles epidemic model by incorporating the Caputo-type nonlocal operator. The objective is to assess its performance against the traditional classical model using real-world data. The results reveal that in scenarios where the dataset exhibits a consistent upward or downward trend, the fractional model outperforms its classical counterpart. This deduction is made by considering the reduced residual error values achieved using a nonlinear least squares curve-fitting technique. Furthermore, Qureshi and Jan [32] created a compartmental model using fractional calculus to analyze the spread of measles in the context of vaccination. According to their study, the most effective approach to tackling measles involves the appropriate utilization of vaccination. Qureshi and Jan [32] further developed a compartmental model within the fractional framework to examine how memory affects the transmission dynamics of measles, incorporating vaccination. Meanwhile, Nazir et al. [64] provided an analytical resolution for a model addressing the propagation of measles through three rounds of vaccination, utilizing the Caputo–Fabrizio fractional derivative (CFFD). Meanwhile, Momani et al. [65] formulated a model for measles infection, considering the impact of vaccination using FO principles. It is noteworthy that, while a significant amount of research has been carried out on the transmission and control of measles, there has not been a comprehensive exploration of the effects of vaccination and quarantine on measles transmission. This current work seeks to formulate a fractional-order model for the transmission dynamics of measles incorporating vaccination and quarantine. We apply the novel Jacobian determinant method [66] to calculate the basic reproduction number of the system and ascertain that it plays a crucial role in determining disease dynamics.

The remainder of the work follows this structure. In the next section of this work, we will present some useful definitions of fractional calculus. We will then present the model formulation and its corresponding analysis in Sections 3 and 4, respectively. In addition, in Section 5, we will conduct numerical simulations for the nonlinear Caputo fractional-order dynamic model that we will construct in Section 3 using the Adams-type predictor-corrector iterative scheme studied in to perform numerical simulations for the nonlinear Caputo fractional-order dynamic model that would be constructed in [67, 68]. Finally, the concluding remarks and future extensions of our study will be provided in Section 6.

## 2. Preliminaries

In this section, we provide some basic definitions and properties of fractional derivatives.

**Definition 1 .**

(see [69, 70]) Fractional integral of order *α* is defined by

(1) $$\begin{array}{c} I^{\alpha }u\left(t\right)=\frac{1}{\Gamma \left(\alpha \right)}\overset{t}{\underset{0}{\int }}\frac{u\left(x\right)}{{\left(t-x\right)}^{1-\alpha }}dx, \end{array}$$

for 0 < *α* < 1, *t* > 0.

**Definition 2 .**

(see [69, 70]) Caputo fractional integral of order *α* is defined by

(2) $$\begin{array}{c} D^{\alpha }u\left(t\right)=\frac{1}{\Gamma \left(q-\alpha \right)}\overset{t}{\underset{0}{\int }}\frac{u^{q}\left(x\right)}{{\left(t-x\right)}^{\alpha +1-q}}dx, \end{array}$$

for *q* − 1 < *α* < *q*.

**Theorem 3 (see [71]).**

The Caputo fractional derivative transformed by Laplace with *u*(*t*) as the function is defined by

(3) LDtCααut=sαUs−∑k=0n−1uk0sα−k−1,

where *n* − 1 < *α* < *n* and *n* ∈ *ℕ*.

**Theorem 4 (see [69]).**

“Mittag–Leffler functions with one parametric and two parametric are given as follows: *E*_*a*_1__(*s*) = ∑_*i*=0_^*∞*^(*s*^*i*^/Γ(*a*_1_*i* + 1)) and *E*_*a*_1_,*a*_2__(*s*) = ∑_*i*=0_^*∞*^(*s*^*i*^/Γ(*a*_1_*i* + *a*_2_)), where *a*_1_, *a*_2_ ∈ *ℝ*^+^.”

**Lemma 5 (see [13]).**

“Let 0 < *α* ≤ 1, *u*(*t*) ∈ *C*[*p*, *q*] and if ^*C*^*D*_*t*_^*α*^*u*(*t*) is continuous in [*p*, *q*], then *u*(*x*) = *u*(*p*) + (1/Γ*α*)(*x* − *p*)^*α*^.^*C*^*D*_*t*_^*α*^*u*(*s*), where 0 ≤ *s* ≤ *x*, ∀*x* ∈ (*p*, *q*].”

Note “*u*(*t*) is a nondecreasing (nonincreasing) function for *t* ∈ [*p*, *q*], if ^*C*^*D*_*t*_^*α*^*u*(*t*) ≥ 0(^*C*^*D*_*t*_^*α*^*u*(*t*) ≤ 0), *t* ∈ (*p*, *q*).”

**Lemma 6 (see [13]).**

“Considering the fractional-order system as”

(4) DtαCXt=ΨX,Xt0=xt01,xt02,xt03…,xt0n,xt0j, j=1,2,…,n,

with 0 < *α* < 1, *X*(*t*) = (*x*^1^(*t*), *x*^2^(*t*),…, *x*^*n*^(*t*)), and Ψ(*X*) : [*t*_0_, *∞*]⟶*ℝ*^*n*×*n*^. Calculating the equilibria, Ψ(*X*) = 0. The equilibria are locally asymptotically stable if and only if each eigen value *λ*_*j*_ of the Jacobian matrix *J*(*X*) = (*∂*(Ψ_1_, Ψ_2_,…, Ψ_*n*_)/*∂*(*x*^1^, *x*^2^,…, *x*^*n*^)) calculated at the equilibria satisfies |arg(*λ*_*j*_)| < (*απ*^*α*^/2).

**Lemma 7 (see [13]).**

If *u*(*t*) ∈ *ℝ*^+^ is a differentiable function, then for any *t* < 0,

(5) DtαCut−u∗−u∗ lnutu∗≤1−u∗utDtαCut, u∗∈R+,∀α∈0,1″.

## 3. Model Formulation

This section presents a fractional-order system for measles. The total human population is divided into six classes. The total population is represented as *N*(*t*), which is made up of the susceptible (*S*), exposed, vaccinated (*V*), infected (*I*), quarantined (*Q*), and recovered classes, respectively.

(6) $$\begin{array}{c} \text{Thus }N\left(t\right)=S\left(t\right)+E\left(t\right)+V\left(t\right)+I\left(t\right)+Q\left(t\right)+R\left(t\right). \end{array}$$

The susceptible population grows with the rate of *ω*, which includes new births and people moving in. Vaccination occurs at a rate of *b*, but vaccinated people may lose immunity (either naturally or due to vaccine failure) at a rate of *τ* and become susceptible again. The probability of infection for susceptible individuals in contact with infected individuals is *βSI*, where *β* is the transmission probability. The transition rate from the exposed to infectious stage is *μ*. Infected individuals are isolated for treatment at a rate of *η*. The recovery rates for infected and quarantined individuals are *θ* and *δ*, respectively. The death rate due to natural causes is *π*, while the measles-induced death rates for infected and quarantined individuals are *c* and *f*, respectively. The nonlinear fractional-order system describing the dynamics of the model is

(7) DtαCSt=ω+τV−b+π+βIS;DtαCEt=βIS−π+μE;DtαCVt=bS−π+τV;DtαCIt=μE−f+π+η+θI;DtαCQt=ηI−c+π+δQ;DtαCRt=θI+δQ−πR;

with non-negative initial conditions *S*(0) = *S*_0_ > 0, *E*(0) = *E*_0_ > 0, *V*(0) = *V*_0_ > 0, *I*(0) = *I*_0_ > 0, *Q*(0) = *Q*_0_ > 0, and *R*(0) = *R*_0_ > 0.

Here, *α* ∈ (0, 1] represents the order of the fractional derivative. When *α* = 1, then the model is the integer order counterpart. The fractional derivative of the model in (7) is used in the Caputo sense, i.e.,

(8) $$\begin{array}{c} \frac{d^{\alpha }y\left(t\right)}{dt^{\alpha }}=I^{q-\alpha }y^{\left(q\right)},\;t>0, \end{array}$$

where *q* = [*α*] is the value of *α* rounded up to the nearest integer, *y*^(*q*)^ is the *q*-th derivative of *y*(*r*), and *I*^*q*_1_^ is the Riemann–Liouville fractional integral given by

(9) $$\begin{array}{c} I^{q_{1}}z\left(t\right)=\frac{1}{\Gamma \left(q_{1}\right)}\overset{t}{\underset{0}{\int }}{\left(t-t^{\prime }\right)}^{q_{1}-1}z\left(t^{\prime }\right)dt^{\prime }, \end{array}$$

where Γ(*q*_1_) is the gamma function.

The model in (7) exhibits some problems in what concerns time dimension between left- and right-hand sides of the equations. On the left-hand side, the dimension is (time)^−*α*^, whereas on the right-hand side, the dimension is (time)^−1^. The corrected system corresponding to the model in (7) is written as [72]

(10) DtαCSt=ωα+ταV−bα+πα+βαIS;DtαCEt=βαIS−πα+μαE;DtαCVt=bαS−πα+ταV;DtαCIt=μαE−fα+πα+ηα+θαI;DtαCQt=ηαI−cα+πα+δαQ;DtαCRt=θαI+δαQ−παR.

## 4. Model Analysis

### 4.1. Non-Negativity and Boundedness of the Model

**Theorem 8 .**

The closed set

(11) $$\begin{array}{c} \Omega =\left\{\left(S,E,V,I,Q,R\right)\in R_{+}^{6}\left|0\right.\leq N\left(t\right)\leq \frac{\omega^{\alpha }}{\pi^{\alpha }}\right\}, \end{array}$$

is positively invariant for the fractional system (10) ∀*t*≥0.

**Proof**

Adding all the equations, we have

(12) DtαCS+E+V+I+Q+Rt=ωα−παS+E+V+I+Q+Rt−fαIt−cαQt.

This implies

(13) DtαCNt=ωα−παNt−Ifα−Qcα.

In the absence of the disease (measles), *I* = *Q* = 0, equation (13) becomes

(14) DtαCNt=ωα−παNt,

which gives

(15) DtαCNt+παNt=ωα.

Applying Laplace transform and Theorem 7.2 in [73], we get

(16) $$\begin{array}{c} s^{\alpha }L\left(N\left(t\right)\right)-s^{\alpha -1}N\left(0\right)+\pi^{\alpha }L\left(N\left(t\right)\right)=\frac{\omega^{\alpha }}{s^{\alpha }}, \end{array}$$

and this implies

(17) $$\begin{array}{c} L\left(N\left(t\right)\right)\left(s^{\alpha +1}+\pi^{\alpha }\right)=s^{\alpha }N\left(0\right)+\omega^{\alpha },\\ L\left(N\left(0\right)\right)=\frac{s^{\alpha }N\left(0\right)+\omega^{\alpha }}{s^{\alpha +1}+\pi^{\alpha }}=\frac{s^{\alpha }N\left(0\right)}{s^{\alpha +1}+\pi^{\alpha }}+\frac{\omega^{\alpha }}{s^{\alpha +1}+\pi^{\alpha }}. \end{array}$$

Using inverse Laplace transform gives

(18) $$\begin{array}{c} N\left(t\right)=N\left(0\right)E_{\alpha ,1}\left(-\pi^{\alpha }t^{\alpha }\right)+\omega^{\alpha }t^{\alpha }E_{\alpha ,\alpha +1}\left(-\pi^{\alpha }t^{\alpha }\right). \end{array}$$

Based on the Mittag–Leffler function,

(19) $$\begin{array}{c} E_{a,b}\left(x\right)=xE_{a,a+b}\left(x\right)+\frac{1}{\Gamma \left(b\right)}. \end{array}$$

Therefore,

(20) $$\begin{array}{c} N\left(t\right)=\left(N\left(0\right)-\frac{\omega^{\alpha }}{\pi^{\alpha }}\right)E_{\alpha ,1}\left(-\pi^{\alpha }t^{\alpha }\right)+\frac{\omega^{\alpha }}{\pi^{\alpha }}. \end{array}$$

Hence,

(21) $$\begin{array}{c} \lim_{t⟶\infty }\mathrm{Sup}\;N\left(t\right)\leq \frac{\omega^{\alpha }}{\pi^{\alpha }}. \end{array}$$

Therefore, the region *Ω* defined by

(22) $$\begin{array}{c} \Omega =\left\{\left(S,E,V,I,Q,R\right)\in R_{+}^{6}\left|0\right.\leq N\left(t\right)\leq \frac{\omega^{\alpha }}{\pi^{\alpha }}\right\}, \end{array}$$

is an attractor of the model, such that all solution curves that begin in *ω*^*α*^ remain in it for all future times.

### 4.2. Existence and Uniqueness

The necessary and sufficient conditions for the existence and uniqueness of the solution to the FO model are outlined in the following theorem and its corresponding proof.

**Theorem 9 .**

There exist a unique solution of the FO model (5) based on each initial condition.

**Proof**

Searching for a condition that is adequate to ensure the existence and uniqueness of the solution for the model in (5) within the specified region *Υ* × (0, *𝒯*] where

(23) $$\begin{array}{c} \Upsilon =\left\{\left(S,E,V,I,Q,R\right)\in R^{6}:\;\max\left\|S\right\|,\left\|E\right\|,\left\|V\right\|,\left\|I\right\|,\left\|Q\right\|,\left\|R\right\|\leq M\right\}. \end{array}$$

The method in [74] is used. Taking the mapping *F*(*X*) = (*F*_1_(*X*), *F*_2_(*X*), *F*_3_(*X*), *F*_4_(*X*), *F*_5_(*X*), *F*_6_(*X*), ) where *X* = (*S*, *E*, *V*, *I*, *Q*, *R*) and $\overset{¯}{X}=\left(\overset{¯}{S},\overset{¯}{E},\overset{¯}{V},\overset{¯}{I},\overset{¯}{Q},\overset{¯}{R}\right)$,

(24) $$\begin{array}{c} F_{1}\left(X\right)=\omega^{\alpha }+\tau^{\alpha }V\left(t\right)-\left(b^{\alpha }+\pi^{\alpha }+\beta^{\alpha }I\left(t\right)\right)S\left(t\right),\\ F_{2}\left(X\right)=\beta^{\alpha }S\left(t\right)I\left(t\right)-\left(\pi^{\alpha }+\mu^{\alpha }\right)E\left(t\right),\\ F_{3}\left(X\right)=b^{\alpha }S\left(t\right)-\left(\pi^{\alpha }+\tau^{\alpha }\right)V\left(t\right),\\ F_{4}\left(X\right)=\mu^{\alpha }E\left(t\right)-\left(f^{\alpha }+\pi^{\alpha }+\theta^{\alpha }+\eta^{\alpha }\right)I\left(t\right),\\ F_{5}\left(X\right)=\eta^{\alpha }I\left(t\right)-\left(c^{\alpha }+\pi^{\alpha }+\delta^{\alpha }\right)Q\left(t\right),\\ F_{6}\left(X\right)=\theta^{\alpha }I\left(t\right)+\delta^{\alpha }Q-\pi^{\alpha }R\left(t\right). \end{array}$$

For any *X*, $\overset{¯}{X}\in \Upsilon$;

(25) $$\begin{array}{c} \left\|F\left(X\right)-F\left(\overset{¯}{X}\right)\right\|=\left|F_{1}\left(X\right)-F_{1}\left(\overset{¯}{X}\right)\right|+\left|F_{2}\left(X\right)-F_{2}\left(\overset{¯}{X}\right)\right|+\left|F_{3}\left(X\right)-F_{3}\left(\overset{¯}{X}\right)\right|+\left|F_{4}\left(X\right)-F_{4}\left(\overset{¯}{X}\right)\right|+\left|F_{5}\left(X\right)-F_{5}\left(\overset{¯}{X}\right)\right|+\left|F_{6}\left(X\right)-F_{6}\left(\overset{¯}{X}\right)\right|\\ =\left|\omega^{\alpha }+\tau^{\alpha }V\left(t\right)-\beta^{\alpha }S\left(t\right)I\left(t\right)-\left(b+\pi^{\alpha }\right)S\left(t\right)-\omega^{\alpha }-\tau^{\alpha }\overset{¯}{V}\left(t\right)+\beta^{\alpha }\overset{¯}{S}\left(t\right)\overset{¯}{I}\left(t\right)+\left(b^{\alpha }+\pi^{\alpha }\right)\overset{¯}{S}\left(t\right)\right|\\ +\left|\beta^{\alpha }S\left(t\right)I\left(t\right)-\left(\mu^{\alpha }+\pi^{\alpha }\right)E\left(t\right)-\beta^{\alpha }\overset{¯}{S}\left(t\right)\overset{¯}{I}\left(t\right)+\left(\mu^{\alpha }+\pi^{\alpha }\right)\overset{¯}{E}\left(t\right)\right|+\left|b^{\alpha }S\left(t\right)-\left(\pi^{\alpha }+\tau^{\alpha }\right)V\left(t\right)-b^{\alpha }\overset{¯}{S}\left(t\right)+\left(\pi^{\alpha }+\tau^{\alpha }\right)\overset{¯}{V}\left(t\right)\right|\left.+\right|\mu^{\alpha }E\left(t\right)-\left(f^{\alpha }+\pi^{\alpha }+\theta^{\alpha }+\eta^{\alpha }\right)I\left(t\right)-\mu^{\alpha }\overset{¯}{E}\left(t\right)+\left(f^{\alpha }+\pi^{\alpha }+\theta^{\alpha }+\eta^{\alpha }\right)\overset{¯}{I}\left(t\right)\left|+\right|\eta^{\alpha }I\left(t\right)\\ -\left(c^{\alpha }+\pi^{\alpha }+\delta^{\alpha }\right)Q\left(t\right)-\eta^{\alpha }\overset{¯}{I}\left(t\right)+\left(c+\pi^{\alpha }+\delta^{\alpha }\right)\overset{¯}{Q}\left(t\right)\left|\;\right.+\left|\theta^{\alpha }I\left(t\right)+\delta^{\alpha }Q\left(t\right)-\pi^{\alpha }R\left(t\right)-\theta^{\alpha }\overset{¯}{I}\left(t\right)-\delta^{\alpha }\overset{¯}{Q}\left(t\right)+\pi^{\alpha }\overset{¯}{R}\left(t\right)\right|,\\ =\left|\tau^{\alpha }\left(V\left(t\right)-\overset{¯}{V}\left(t\right)\right)+\beta^{\alpha }\left(\overset{¯}{S}\left(t\right)\overset{¯}{I}\left(t\right)-S\left(t\right)I\left(t\right)\right)+\left(b^{\alpha }+\pi^{\alpha }\right)\left(\overset{¯}{S}\left(t\right)-S\left(t\right)\right)\right|+\left|\beta^{\alpha }\left(S\left(t\right)I\left(t\right)-\overset{¯}{S}\left(t\right)\overset{¯}{I}\left(t\right)\right)+\left(\mu^{\alpha }+\pi^{\alpha }\right)\left(\overset{¯}{E}\left(t\right)-E\left(t\right)\right)\right|+\left|b^{\alpha }\left(S\left(t\right)-\overset{¯}{S}\left(t\right)\right)+\left(\pi^{\alpha }+\tau^{\alpha }\right)\left(\overset{¯}{V}\left(t\right)-V\left(t\right)\right)\right|\\ +\left|\mu^{\alpha }\left(E\left(t\right)-\overset{¯}{E}\left(t\right)\right)+\left(f^{\alpha }+\pi^{\alpha }+\theta^{\alpha }+\eta^{\alpha }\right)\left(\overset{¯}{I}\left(t\right)-I\left(t\right)\right)\right|+\left|\eta^{\alpha }\left(I\left(t\right)-\overset{¯}{I}\left(t\right)\right)+\left(c^{\alpha }+\pi^{\alpha }+\delta^{\alpha }\right)\left(\overset{¯}{Q}\left(t\right)-Q\left(t\right)\right)\right|+\left|\theta^{\alpha }\left(I\left(t\right)-\overset{¯}{I}\left(t\right)\right)+\delta^{\alpha }\left(Q\left(t\right)-\overset{¯}{Q}\left(t\right)\right)+\pi^{\alpha }\left(\overset{¯}{R}\left(t\right)-R\left(t\right)\right)\right|,\\ \leq \left(2\beta^{\alpha }+\left(2b^{\alpha }+\pi^{\alpha }\right)M\right)\left|\left(\overset{¯}{S}\left(t\right)-S\left(t\right)\right)\right.\left|+\left(2\mu^{\alpha }+\pi^{\alpha }\right)\right|\left(E\left(t\right)-\overset{¯}{E}\left(t\right)\right)\left|\;\right.\left.+\left(\pi^{\alpha }+2\tau^{\alpha }\right)\right|\left(\overset{¯}{V}\left(t\right)-V\left(t\right)\right)+\left(f^{\alpha }+\pi^{\alpha }+2\theta^{\alpha }+2\eta^{\alpha }\right)\left|\left(\overset{¯}{I}\left(t\right)-I\left(t\right)\right)\right|+\left(c^{\alpha }+\pi^{\alpha }+2\delta^{\alpha }\right)\left|\left(\overset{¯}{Q}\left(t\right)-Q\left(t\right)\right)\right|+\pi^{\alpha }\left|\left(\overset{¯}{R}\left(t\right)-R\left(t\right)\right)\right|,\\ \leq P_{1}\left|S-\overset{¯}{S}\right|+P_{2}\left|E-\overset{¯}{E}\right|+P_{3}\left|V-\overset{¯}{V}\right|+P_{4}\left|I-\overset{¯}{I}\right|+P_{5}\left|Q-\overset{¯}{Q}\right|+P_{6}\left|R-\overset{¯}{R}\right|\leq P\left\|X-\overset{¯}{X}\right\|. \end{array}$$

where *P* = max {*P*_1_, *P*_2_, *P*_3_, *P*_4_, *P*_5_,  and *P*_6_} and *P*_1_ = (2*β*^*α*^ + (2*b*^*α*^ + *π*^*α*^)*M*), *P*_2_ = (2*μ*^*α*^ + *π*^*α*^), *P*_3_ = (*π*^*α*^ + 2*τ*^*α*^), *P*_4_ = (*f*^*α*^ + *π*^*α*^ + 2*θ*^*α*^ + 2*η*^*α*^),  and *P*_5_ = (*c* + *π*^*α*^ + 2*δ*^*α*^), *P*_6_ = *π*^*α*^.

Therefore, *F*(*X*) fulfils the Lipschitz condition. As a result, the FO model (10) exists and is unique.

### 4.3. Equilibria of the Model

The model in (10) has two equilibrium points: the measles-free equilibrium given by

(26) $$\begin{array}{c} E_{0}=\left(S^{0}=\frac{\omega^{\alpha }\;\left(\pi^{\alpha }+\tau^{\alpha }\right)}{\pi^{\alpha }\;\left(b^{\alpha }+\pi^{\alpha }+\tau^{\alpha }\right)},0,V^{0}=\frac{b^{\alpha }\omega^{\alpha }}{\pi^{\alpha }\left(b^{\alpha }+\pi^{\alpha }+\tau^{\alpha }\right)},0,0,0\right). \end{array}$$

The Jacobian determinant method [66] is used to calculate the threshold quantity (the basic reproductive number) of the model in (10). The Jacobian determinant model of the infected subcompartment evaluated at the measles-free equilibrium *ℰ*_0_ is given by

(27) $$\begin{array}{c} J\left(E_{0}\right)=\left[\begin{array}{cc} -\left(\pi^{\alpha }+\mu^{\alpha }\right) & \frac{\beta^{\alpha }\omega^{\alpha }\left(\pi^{\alpha }+\tau^{\alpha }\right)}{\pi^{\alpha }\;\left(b^{\alpha }+\pi^{\alpha }+\tau^{\alpha }\right)}\\ \mu^{\alpha } & -\left(f^{\alpha }+\pi^{\alpha }+\eta^{\alpha }+\theta^{\alpha }\right) \end{array}\right], \end{array}$$

whose determinant is given by

(28) $$\begin{array}{c} \left|J\left(E_{0}\right)\right|=\left(\pi^{\alpha }+\mu^{\alpha }\right)\left(f^{\alpha }+\pi^{\alpha }+\eta^{\alpha }+\theta^{\alpha }\right)-\frac{\beta^{\alpha }\omega^{\alpha }\mu^{\alpha }\left(\pi^{\alpha }+\tau^{\alpha }\right)}{\pi^{\alpha }\left(b^{\alpha }+\pi^{\alpha }+\tau^{\alpha }\right)}, \end{array}$$

which can be written as

(29) $$\begin{array}{c} \left|J\left(E_{0}\right)\right|=-\left(\pi^{\alpha }+\mu^{\alpha }\right)\left(f^{\alpha }+\pi^{\alpha }+\eta^{\alpha }+\theta^{\alpha }\right)\left[\frac{\beta^{\alpha }\mu^{\alpha }\omega^{\alpha }\left(\pi^{\alpha }+\tau^{\alpha }\right)}{\pi^{\alpha }\;\left(b^{\alpha }+\pi^{\alpha }+\tau^{\alpha }\right)\left(\pi^{\alpha }+\mu^{\alpha }\right)\left(f^{\alpha }+\pi^{\alpha }+\eta^{\alpha }+\theta^{\alpha }\right)}-1\right]. \end{array}$$

Therefore, the threshold quantity is given by

(30) $$\begin{array}{c} R_{0}^{\prime }=\frac{\beta^{\alpha }\mu^{\alpha }\omega^{\alpha }\left(\pi^{\alpha }+\tau^{\alpha }\right)}{\pi^{\alpha }\left(b^{\alpha }+\pi^{\alpha }+\tau^{\alpha }\right)\left(\pi^{\alpha }+\mu^{\alpha }\right)\left(f^{\alpha }+\pi^{\alpha }+\eta^{\alpha }+\theta^{\alpha }\right)}. \end{array}$$

The measles-endemic equilibrium is *ℰ*^∗^ = (*S*^∗^, *E*^∗^, *V*^∗^, *I*^∗^, *Q*^∗^, *R*^∗^), where

(31) $$\begin{array}{c} \left.\begin{array}{c} S^{*}=\frac{\omega^{\alpha }\left(\pi^{\alpha }+\tau^{\alpha }\right)}{\pi^{\alpha }\;\left(b^{\alpha }+\pi^{\alpha }+\tau^{\alpha }\right)+\left(\pi^{\alpha }+\tau^{\alpha }\right)\beta^{\alpha }I^{*}};\\ E^{*}=\frac{\beta^{\alpha }\;\omega^{\alpha }\left(\pi^{\alpha }+\tau^{\alpha }\right)I^{*}}{\left(\pi^{\alpha }\;\left(b^{\alpha }+\pi^{\alpha }+\tau^{\alpha }\right)+\left(\pi^{\alpha }+\tau^{\alpha }\right)\beta^{\alpha }I^{*}\right)\left(\pi^{\alpha }+\mu^{\alpha }\right)};\\ V^{*}=\frac{b^{\alpha }\omega^{\alpha }}{\pi^{\alpha }\;\left(b^{\alpha }+\pi^{\alpha }+\tau^{\alpha }\right)+\left(\pi^{\alpha }+\tau^{\alpha }\right)\beta^{\alpha }I^{*}};\\ I^{*}=\frac{\mu^{\alpha }E^{*}}{\left(f^{\alpha }+\pi^{\alpha }+\eta^{\alpha }+\theta^{\alpha }\right)};\\ Q^{*}=\frac{\eta^{\alpha }I^{*}}{\left(c^{\alpha }+\pi^{\alpha }+\delta^{\alpha }\right)};\\ R^{*}=\frac{\left(\theta^{\alpha }\left(c+\pi^{\alpha }\right)+\left(\theta^{\alpha }+\eta^{\alpha }\right)\delta^{\alpha }\right)I^{*}}{\left(c+\pi^{\alpha }+\delta^{\alpha }\right)\pi^{\alpha }}. \end{array}\right\} \end{array}$$

### 4.4. Stability Analysis of the Model at *ℰ*_0_

**Theorem 10 .**

When *ℛ*_0_ < 1, the model in (5) is GAS, and unstable when *ℛ*_0_ > 1 at *ℰ*_0_.

**Proof**

Applying the right Lyapunov function's time derivative,

(32) DtαCFt=μαE+πα+μαI.

The abovementioned function's time derivative is

(33) DtαCFt=μαCDtαEt+πα+μαCDtαIt.

From (7), we obtain

(34) DtαCFt=μαβαIS−πα+μαE+πα+μαμαE−fα+πα+ηα+θαI,DtαCFt=μαβαIS−πα+μαfα+πα+ηα+θαI=Iπα+μαfα+πα+ηα+θαμαβαSπα+μαfα+πα+ηα+θα−1=Iπα+μαfα+πα+ηα+θαR0−1.

Therefore, if *ℛ*_0_ < 1, then ^*C*^*D*_*t*_^*α*^*ℱ*(*t*) < 0.

As the outcome of LaSalle's use of Lyapunov's concept [75, 76], the point *ℰ*_0_ is GAS and unstable if *ℛ*_0_ > 1.

### 4.5. Stability Analysis of the Model at *ℰ*^∗^

**Theorem 11 .**

The model in (5) is GAS at *ℰ*^∗^*ifℛ*_0_ > 1.

**Proof**

The Lyapunov function in the Goh–Volterra form is outlined as follows:

(35) $$\begin{array}{c} K=\left(S-S^{*}-S^{*}\;\log\frac{S}{S^{*}}\right)+\left(E-E^{*}-E^{*}\;\log\frac{E}{E^{*}}\right)+L\left(I-I^{*}-I^{*}\;\log\frac{I}{I^{*}}\right). \end{array}$$

Applying Lemma 7 and Caputo derivative, gives

(36) DtαCKt≤1−S∗SDtαCSt+1−E∗EDtαCEt+L1−I∗IDtαCIt.

Applying (7), gives

(37) DtαCKt≤ωα−bα+παS−βαSI−S∗ωα−bα+παS−βαSIS+βαIS−πα+μαE−E∗βαIS−πα+μαEE+LμαE−fα+πα+ηα+θαI−I∗μαE−fα+πα+ηα+θαI.

Equation (7) gives the equilibrium point,

(38) $$\begin{array}{c} \omega^{\alpha }=\left(b^{\alpha }+\pi^{\alpha }\right)S^{*}+\beta^{\alpha }S^{*}I^{*}. \end{array}$$

Putting (38) into (37) gives

(39) DtαCKt≤bα+παS∗+βαS∗I∗+ταV−bα+παS−βαSI−S∗bα+παS∗+βαS∗I∗−bα+παS−βαSIS+βαIS−πα+μαE−E∗βαIS−πα+μαEE+LμαE−fα+πα+ηα+θαI−I∗μαE−fα+πα+ηα+θαI.

Further simplification gives

(40) DtαCKt≤bα+παS∗+βαS∗I∗−bα+παS−S∗bα+παS∗+βαS∗I∗−bα+παS−βαSIS+−πα+μαE−E∗βαIS−πα+μαEE+LμαE−f+πα+ηα+θαI−I∗μαE−fα+πα+ηα+θαI.

Consider all infected classes that do not contain a single star (∗) from (40) and set them equal to zero:

(41) $$\begin{array}{c} \beta^{\alpha }S^{*}-\left(\pi^{\alpha }+\mu^{\alpha }\right)E+L\left(\mu^{\alpha }E-\left(f^{\alpha }+\pi^{\alpha }+\eta^{\alpha }+\theta^{\alpha }\right)I\right)=0. \end{array}$$

The equilibrium point was slightly perturbed between (7) and (41) with outcome as

(42) $$\begin{array}{c} L=\frac{\beta^{\alpha }S^{*}}{\left(f^{\alpha }+\pi^{\alpha }+\eta^{\alpha }+\theta^{\alpha }\right)},\\ \left(\mu^{\alpha }+\pi^{\alpha }\right)=\frac{\beta^{\alpha }S^{*}I^{*}}{E^{*}},\\ \mu^{\alpha }=\frac{\left(f^{\alpha }+\pi^{\alpha }+\eta^{\alpha }+\theta^{\alpha }\right)I^{*}}{E^{*}}. \end{array}$$

Substituting (42) into (40) gives

(43) DtαCKt≤bα+παS∗+βαS∗I∗−bα+παS−S∗βαS∗I∗+bα+παS∗−bα+παSS+−E∗βαSIE+βαS∗I∗+−βαS∗I∗IE∗+βαS∗I∗.

Applying A.M ≥ G.M. gives (2 − (*S*/*S*^∗^) − (*S*^∗^/*S*)) ≤ 0, (3 − (*S*^∗^/*S*) − (*I*^∗^*E*/*IE*^∗^) − (SE^∗^*I*/*E*)) ≤ 0.

Thus, ^*C*^*D*_*t*_^*α*^*K*(*t*) ≤ 0.

Hence, the point *ℰ*^∗^ is GAS if *ℛ*_0_ > 1.

## 5. Numerical Simulations

Numerical solutions for nonlinear dynamic systems are an important aspect of infectious disease epidemiological modeling. In this section, we provide numerical simulations for the measles model (10). We will apply the Adams-type predictor-corrector iterative scheme constructed in [67, 68] to simulate the model problem. For numerical illustrations, we will consider the following initial conditions: *S*(0) = 1000; *E*(0) = 50; *V*(0) = 20; *I*(0) = 5; *Q*(0) = 2; *R*(0) = 0, with parameter values as given in Tables 1 and 2.

**Figure 1:**
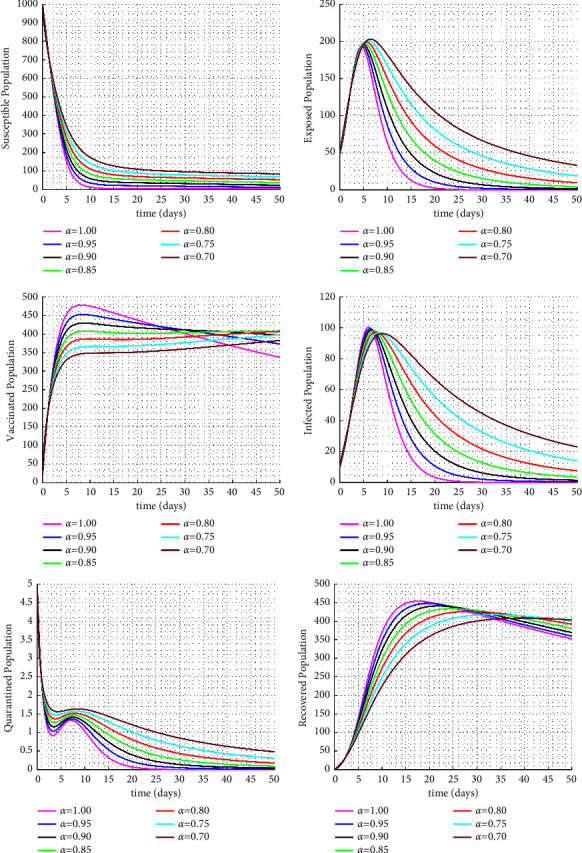
Solution trajectories for the proposed fractional order model with *β* = 0.0038.

**Figure 2:**
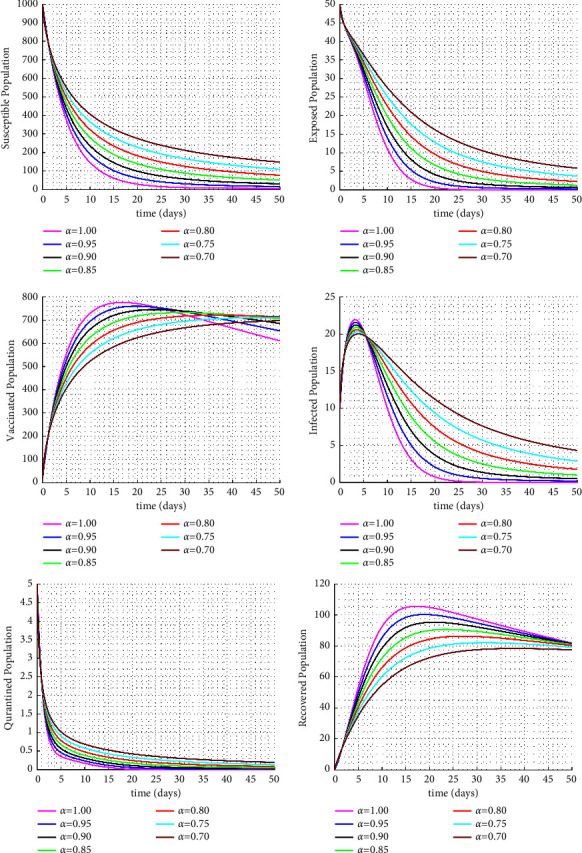
Solution trajectories for the proposed fractional order model with *β* = 0.0008.

Figures 1 and 2 display the fractional trajectories of different classes with varying transmission rates. Figure 1 utilize a transmission rate of *β* = 0.0038, which reveals that an increase in the fractional order leads to a rise in the number of infected individuals, consequently increasing the number of vaccinated, treated, and recovered individuals. On the other hand, the number of susceptible individuals decreases. This suggests that a lack of vaccination can lead to an increase in disease transmission.

**Figure 3:**
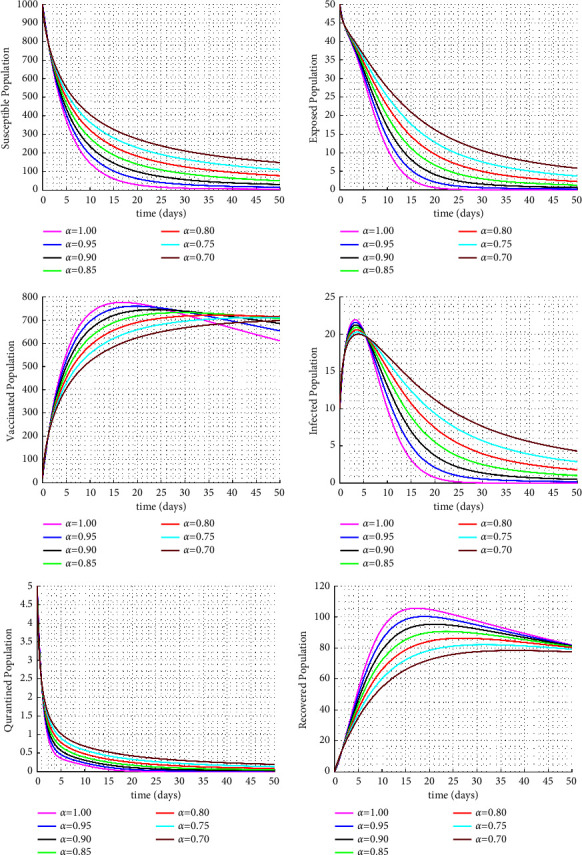
Numerical solutions for the Caputo fractional order dynamic model with isolated rate *η* = 0.012.

**Figure 4:**
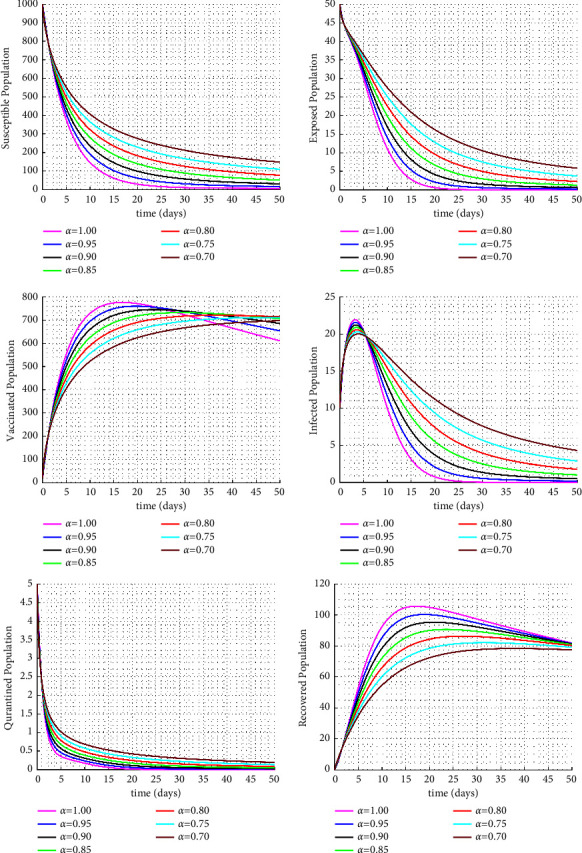
Numerical solutions for the Caputo fractional order dynamic model with isolated rate *η* = 0.112.

**Figure 5:**
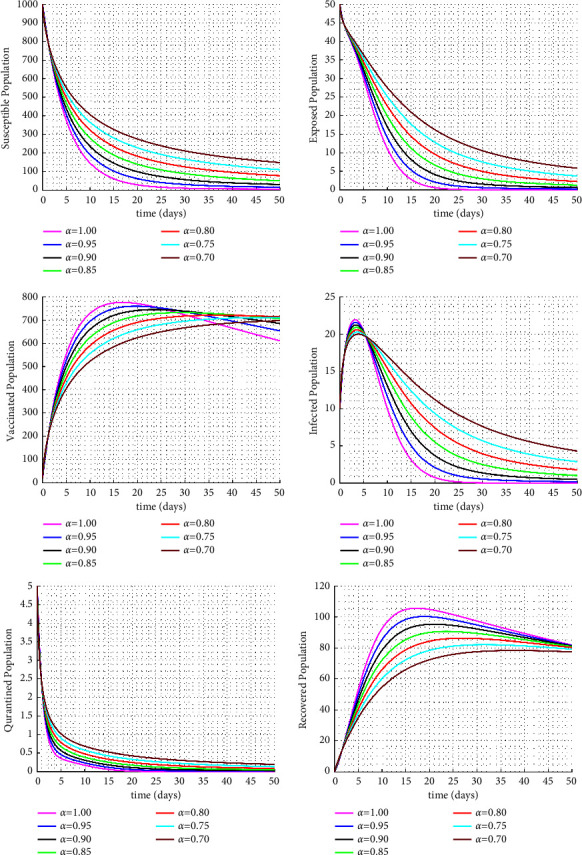
Solution paths for the Caputo fractional-order dynamic model for varying fractional orders.

**Figure 6:**
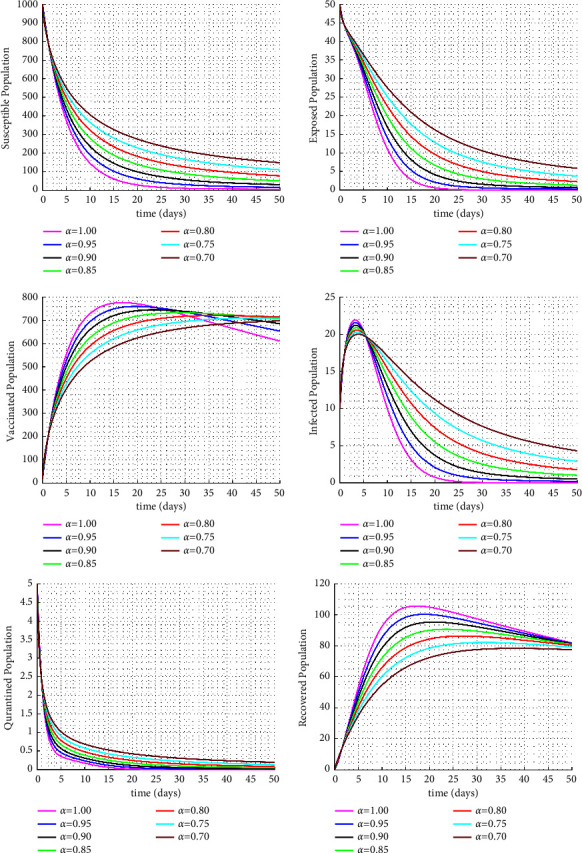
Solution paths for the Caputo fractional order dynamic model for varying fraction orders with vaccinated rate *b*.

Figure 3 examines the impact of the model parameter *η* on the infected class. Increasing the *η* parameter, which represents the isolation rate, results in a significant decrease in the number of infected individuals (see Figures 3, 4 and 5). Furthermore, it can been seen in Figure 6 that as the vaccination rate increases, there is a rapid decrease in the infected class leading to a decrease in the exposed class and an increase in the vaccinated class.

## 6. Conclusions

The present study is concerned with mathematical modeling and analysis of measles infection using the Caputo fractional-order derivative. The mathematical model constructed in this study considers the dynamics of quarantine and vaccination. Using appropriate qualitative techniques, the existence and uniqueness of the solutions of the dynamic model are established. Furthermore, model equilibria are determined, and the new Jacobian determinant method recently developed and studied by the authors in [66] was applied to determine the threshold quantity, *ℛ*_0_. Furthermore, we construct appropriate Lyapunov functions to establish the global asymptotic stability of disease-free and endemic equilibrium points. The findings reveal that if *ℛ*_0_< 1, the point *ℰ*_0_ is GAS. Also, if *ℛ*_0_> 1, the point *ℰ*^∗^ is GAS. From the illustrated numerical simulation results, the present investigation focused on the impact of vaccination and quarantine measures, revealing that they could help limit the spread of measles disease. In future research, we will expand this analysis to include fractal-fractional derivatives, specifically the Caputo fractal-fractional and Atangana–Baleanu fractal-fractional derivative operators. In addition, we will include fractal-fractional dynamics into the optimal control dynamic modeling of this infectious disease.

## Figures and Tables

**Table 1:** State variables for measles fractional model.

State variable	Meaning
*S*	The number of susceptible individuals at time *t*
*E*	The number of exposed individuals (infected but not yet infectious) at time *t*
*V*	The number of vaccinated individuals at time *t*
*I*	The number of infectious individuals at time *t*
*Q*	The number of infected individuals who are quarantined at time *t*
*R*	The number of recovered individuals at time *t*

**Table 2:** System parameter meanings and figures used for simulations.

Parameter	Meaning	Value (per day)	Source
*ω*	Recruitment rate	0.029	Assumed
*η*	The rate at which infected persons are isolated	0.012	Assumed
*c*	The mortality rate of *Q* due to measles disease	0.083	Assumed
*β*	The rate of infection per contact between susceptibles and infectives	0.09091	[77]
*π*	Natural mortality rate	0.0078	[78]
*f*	The mortality rate of *I* due to measles disease	0.125	[30]
*τ*	Immunity decay rate	0.0017	Assumed
*b*	The rate at which susceptibles are vaccinated	0.7	[28]
*θ*	The recovery rate of *I* following treatment	0.14286	[79]
*δ*	The rate of recovery of *Q* following treatment	0.023	Assumed
*μ*	The rate of progression of *E* class to *I* class	0.125	[77]

## Data Availability

All the data used to support the findings of this study are included within the article.
